# Effective Design of Profiling Float Network for Oceanic Heat-Content Monitoring

**DOI:** 10.1155/2014/340518

**Published:** 2014-02-27

**Authors:** Shuhei Masuda, Shigeki Hosoda

**Affiliations:** Research Institute for Global Change, Japan Agency for Marine-Earth Science and Technology (JAMSTEC), 3173-25 Showa-Machi, Kanazawa-Ku, Yokohama, Kanagawa 236-0001, Japan

## Abstract

Schemes for optimizing ocean observation programs are presently the subject of increased interest since in principle they should lead to the improved understanding of the dynamical state of the ocean that is required within the present regime of climate change. Here we use an adjoint sensitivity analysis together with a four-dimensional fluctuating oceanic current system to identify key regions for intensive monitoring by drifting profiling float. In this way we aim to maximize observational efficiency. As a scientific benchmark for the validity of our approach, we have attempted to define the ambient sensitivity of the oceanic heat content to a subtle change in water temperature within the Pacific Basin. We focus on the interannual to multidecadal variations in particular. As a result, sensitivity signals reflecting changes in heat content exhibit a characteristic pattern in the three-dimensional continuum and have drastic temporal changes. This implies that the key regions will depend greatly on the operational timeframe of the observing system. We demonstrate a more effective geographic deployment strategy for the profiling floats monitoring changes in the oceanic heat content on a decadal timescale.

## 1. Introduction

**Figure 1:**
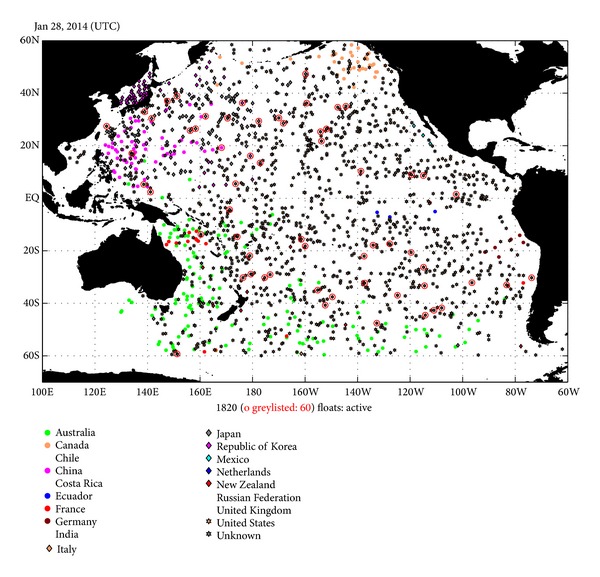
Location of the Argo profiling float observing in the Pacific Ocean active on the 28th of January, 2014. Each location is color-coded by the country of float investigator. Citing from Pacific Argo Regional Center (PARC) (http://www.jamstec.go.jp/ARGORC/location_top.html).

At the beginning of the 2000s, great progress was made in ocean observations with the introduction of Argo profiling floats capable of continuously monitoring ocean properties [1] (Figure 1). Following demonstrative researches which verify the effectiveness of Argo profiling float in the oceanography, strategy of a cost-effective deployment of the instruments is required.

It has been a contemporary interest of oceanographers to construct a practically effective ocean observing system within limited resources. Vecchi and Harrison [2] have investigated the effectiveness of the Indian Ocean Observing System. They examined the impact of the observational frequency and locations on the representation of the water temperature anomaly for a specific subsurface layer by varying the sampling strategy with pseudo-observations from an ocean general circulation model (OGCM). In order to quantitatively assess the impact of these factors, they presented distributions of correlation coefficients between the subsurface temperature anomaly from an OGCM and a reconstructed anomaly derived from subsampled values by XBT lines and Argo arrays.

Although Vecchi and Harrison's approach can provide valuable information that leads to more effective ocean observing systems, the information was limited because it requires a set of independent model calculations based on some ad hoc scenarios. That is why the subsurface depth or sampling strategy is specified in their study. Their approach may therefore be rather favorable to sequential data assimilation, which requires relatively low computational cost.

The adjoint solution of a general circulation model is known to enable us to detect the overall sensitivity of any objective function, for instance, water temperature in a specific location [3] or mass transport through a strait [4], to fluctuations of the model variables. In turn, this facilitates the identification of key regions for intensive monitoring for subtle change in the specific function, which can contribute to an improved ocean state estimation [4].

In this study, we report on adjoint sensitivity analysis using a four-dimensional variational (4D-VAR) ocean data synthesis system that was performed to identify the sensitivity of heat content in the entire Pacific Basin to a water temperature change for a multidecadal timescale. This variable (i.e., heat content) is always of central interest to oceanographers and climate researchers and is particularly relevant to global warming studies [5–7]. We then apply this knowledge to construction of a cost-effective Argo design.

## 2. Methods

A 4D-VAR ocean data synthesis system, developed as a part of the Japan Agency for Marine-Earth Science and Technology (JAMSTEC)-Kyoto University collaborative program (known as “the K7 consortium”) [8, 9] is applied to the adjoint sensitivity analysis, in which the ocean representation moves backwards in time [10, 11]. Adjoint sensitivity analysis here gives the temporal rate of change of an objective function at a fixed point in time when model variables are arbitrarily changed in the 3-dimensional continuum at moments before the fixed point [12, 13].

Objective function *J* is here determined as the heat content integrated from 120°E to 70°W and from 60°S to 60°N over the entire Pacific Basin from surface to bottom. The adjoint model of an OGCM can be used to calculate the sensitivities, ∂*J*/∂*X*(**r**, *t*), where *X* is any variable of concern at location **r** and at time *t*. We are interested in determining how a shift in model variables would affect *J*. We therefore consider the form of *δJ*:

(1) $$\begin{array}{c} \delta J=\frac{\partial J}{\partial X\left(r,t\right)}\delta X\left(r,t\right), \end{array}$$

where *δX* is an estimate of the uncertainty (i.e., the fluctuation of the variable *X*). In this analysis experiment, ∂*X*(**r**, *t*) was represented by a positive value whose magnitude is relevant to a change in subsurface water temperature by 1 Kelvin. This value does not imply any loss of generality. The change is assumed to be expressed as the delta function.

In this study, we made use of an optimized model climatology of the seasonal progression as the background ocean state for the determination of *J*, in the same way as Masuda et al. [9]. The ocean state was validated by using a global map of velocity derived from Argo drift data [14] and an objective analysis result [15]. We performed adjoint sensitivity analysis using the Earth Simulator, JAMSTEC's powerful parallel supercomputer system.

## 3. Results

### 3.1. Sensitivity of Heat-Content

**Figure 2:**
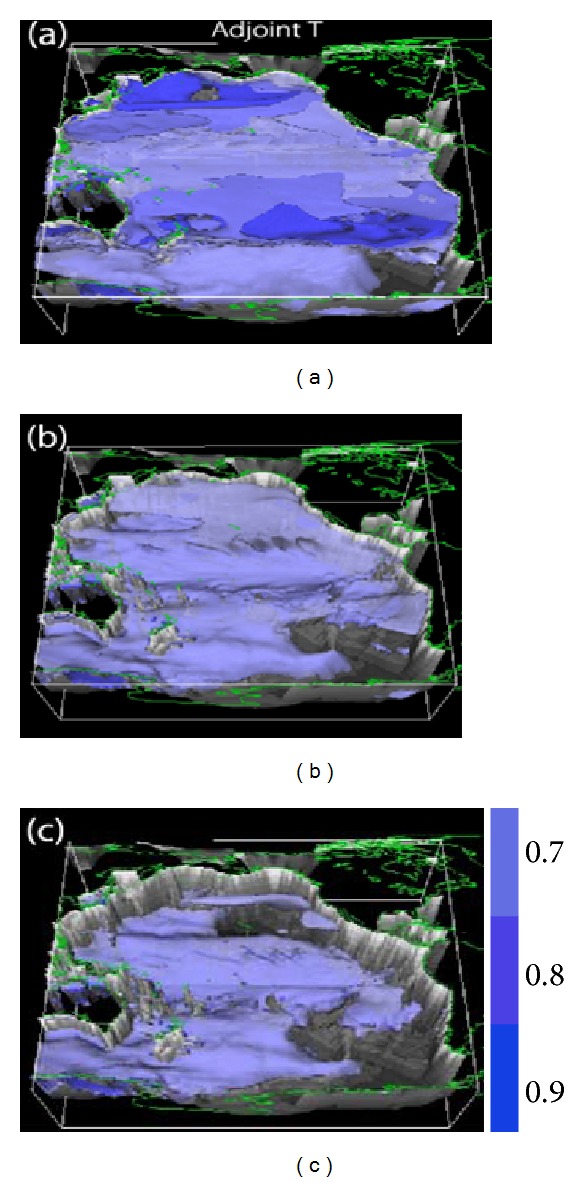
Sensitivity of heat content in the Pacific Ocean at an allocated model time (defined as year zero) when water temperature changes by +1 K at an arbitrary 4-dimensional grid point. The panels show the impact of the change in subsurface water temperature on the heat-content increase after (a) 10 years, (b) 30 years, and (c) 50 years. The units are dimensionless.

Figure 2 shows contour surfaces for the sensitivity, that is, ∂*J*/∂*X*(**r**, *t*), where *J* is the integrated heat content over the entire Pacific Basin and *X* is water temperature. This corresponds to the possible change in water temperature taking place in the entire Pacific Basin at an allocated model time (defined as year zero) when water temperature changes at an arbitrary 3-dimensional grid point on the contour surface over a specific time period (in this case −10, −30, and −50 years, in reverse chronological order). For example, a contour surface of 0.9 at −10 years denotes that a given 1-degree change in water temperature in a specific volume on that surface would possibly cause a heat-content change that is relevant to temperature change by 0.9 degrees in the volume after 10 years. Thus, the value shows how influential the temperature change is in its contribution to the heat-content change after each time period.

The values at shallow depths decrease as the retrospective time increases (Figures 2(a)–2(c)). This is because the relatively strong advective and diffusive effects in the shallow layers tend to spread and redistribute the sensitivity quickly [3]. In contrast, large values tend to persist in the middle depths to the deep ocean. This shows that the influence of change in water temperature on the change in heat content largely depends on the location in the 4-dimensional continuum.

The major distribution of the sensitivity is dominated by the circulation pattern within the first decade. During this stage, the advective effect stands out. Relatively large values are found in the recirculation regions of the horizontal large-scale circulation (Figure 2(a)). The sensitivity is retained in such regions because it tends to be confined by the closed circulation. Subtle wave propagation is another important factor for determining the time-changing distribution. The vigorous wave movement in the equatorial region should quickly redistribute the sensitivity by readily transmitting the information of temperature change in accordance with a given heat-content change to distant locations.

Figure 2(b) shows apparently lower values at the same depth level in the equatorial region between wave guides around 20°N. The wave motion changes the configuration of the isotherms (isopycnals) and thus influences the heat distribution in the targeted volume and thus the aspects of heat input from sea surface.

Figure 2(c) shows that the bottom topography has some impact on the sensitivity distribution. This is due to the interplay between advection and rapid wave propagation in deep layers [9].

**Figure 3:**
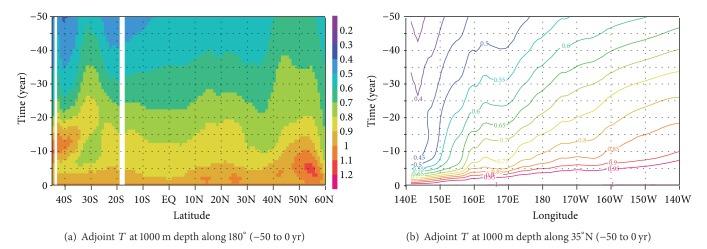
(a) Time-latitude plot of the sensitivities shown in Figure 2, from 45°S to 60°N on the 1000 m isobath along the International Date Line. (b) The same as (a) but for time versus longitude from 140°E to 140°W along 35°N.

We investigated the role of wave motion at the specific depth of 1000 m, which was selected because of its strong relevance to the permanent thermocline in the midlatitude Pacific. The 1000 m isobath, in particular, is also a major drifting depth for Argo floats [1].  Figure 3(a) shows a time-latitude plot of the sensitivity shown in Figure 2 across the entire Pacific Basin along the International Date Line. Note that the vertical axis denotes the retrospective time period. The rate of decrease of the sensitivity values is higher in the equatorial region between 10°S and 10°N. The sensitivity decreases below 0.9 within a 2-year backward calculation. In contrast, it takes about seven years for the sensitivity to decrease below 0.9 at 40°N. This difference comes from the different timescale of the oceanic adjustment, primarily through wave motions [16]. The nonmonotonic changes south of 20°S are caused by the strong influence of advection by the Antarctic Circumpolar Current.

Figure 3(b) shows time-longitude plot of the sensitivity as in Figure 3(a), but along 35°N. It shows the eastward propagation of the sensitivity signal in retrospective time. This indicates a westward propagating ocean adjustment in the real ocean. The propagation speed is largely consistent with that of long Rossby waves reported in the literature of general circulation theory. That is, the major signal propagates across the entire midlatitude Pacific Ocean on a decadal timescale. There seems to be other waves with local effects whose speed of propagation is relatively slow. These “adjustments” in the adjoint calculation are the result of interplay between the higher mode oceanic waves and the background ocean current system, as in the real ocean, but in this case for a backward progression in time.

### 3.2. Effective Argo Design

In this section, we make best use of the adjoint sensitivity to define more effective deployment of the Argo profiling float. The sensitivities described in Section 3.1 show how changes in water temperature in the Pacific regions could impact the targeted climate variation, which in this case was the heat-content change integrated over the entire Pacific Basin. Regions with higher sensitivity values in Figure 3 thus would be key regions to monitor variations of the heat content.

To evaluate the practical importance of each region for the detection of the heat-content change in the Pacific Basin, we have calculated the product of the sensitivity values (Figure 2) and the observation errors. Observational error is difficult to estimate in the subsurface ocean. We adopted the climatological standard deviations as a substitute [4]; these were estimated for each vertical level from a long-term ocean-state estimation by the K7 consortium [7, 9].

**Figure 4:**
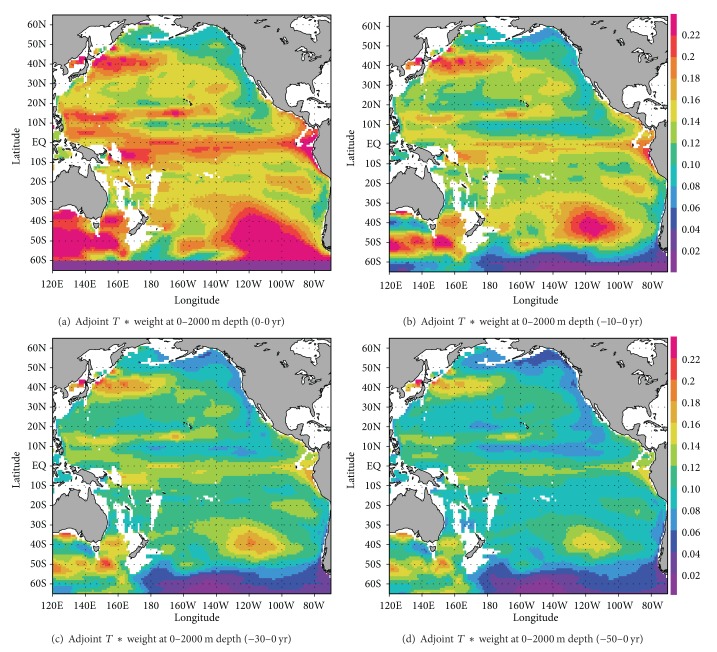
Modified sensitivity calculated as the product of the sensitivity values shown in Figure 2 and the square root of temperature error. The values are averaged over the upper 2000 m of the water column and within specified windows of time. The windows of time are (a) 0 years, (b) 10 years, (c) 30 years, and (d) 50 years (see Section 3.2 in the text for details). The units are in Kelvin.

Figure 4 shows the distribution of the calculated products averaged over the upper 2000 m of the water column and within the allocated model time period. The depth of 2000 m denotes the maximum Argo float depth [1]. The window of time is assumed to be targeted timescale. The geographical patterns show contrast of the representativeness for heat-content change over the relevant window of time. Relatively high positive values are found near the equatorial region, in the central part of the southern subtropical region centered at 40°S, 120°W, and in the western subtropical region along 13°N. The values in the Kuroshio Extension region west of 170°W around 40°N are also relatively high. Such large variabilities highlight the importance of monitoring. This also serves as a reminder that observations in the central part of the southern subtropical region of the southern hemisphere are relatively sporadic at present.

Over a 50-year period, values gradually diminish and the contrast changes region by region. For example, the rate of decay is relatively higher in the equatorial region, according to the temporal evolution of the sensitivity signal (Figure 2). These results imply that regularly arranged deployments of in situ instrumentation are not necessarily the most effective method of monitoring basin-scale oceanic heat-content variations over a multidecadal timescale. A configuration based on the alternative shown in Figure 4 will yield a more effective observation scheme. Note again that the configuration can be adjusted according to the targeted climate variation.

**Figure 5:**
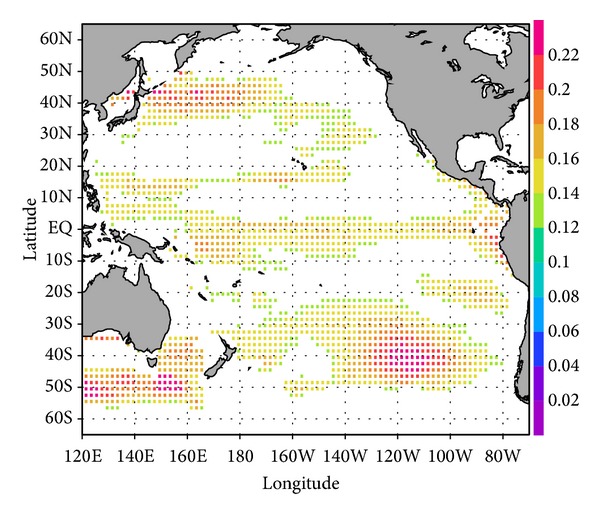
The same as Figure 4(b) but for the 1652 highest sensitivity values (see Section 3.2 in the text for details).

To demonstrate the effectiveness of a systematic deployment deduced from our analyses, we have performed a quantitative assessment for the case of a monitoring program aimed at specifying the decadal evolution of the heat content in the entire Pacific region. We chose two sets of 1652 grid points in a rectangular region of the Pacific Ocean, from 60°S to 60°N and from 120°E to 70°W. This value (1652) corresponds to the number of oceanic observation sites spaced at 3° of latitude by 3° of longitude across the region. The first of these two sets follows a conventional deployment pattern on a regularly assigned grid (i.e., every 3° in both latitude and longitude) [1]. The other represents a systematic deployment consisting of those grid points with the 1652 largest values selected in accordance with the sensitivity shown in Figure 4(b), which were optimally chosen from a regularly assigned grid spaced every 2° in both latitude and longitude (Figure 5).

The total value of the observational sensitivity derived from the “optimal” observing system is 1.21 times that of the conventional one. This implies that intensive deployments in key regions can possibly enhance the accuracy of estimation for the heat-content change in the entire Pacific Basin by 20% for the same resource.

The detailed values can depend on the model platform (e.g., formalism, physical schemes, and resolution) and various assumptions made in an adjoint sensitivity analysis [4, 12]. For instance, the higher resolution systems possibly provide information of a different nature for finer-resolution observations. Nevertheless, the effectiveness of basin-scale strategic deployment at optimally selected sites deduced from the present analysis is clearly defined. The potential of this approach for the design of new global ocean observation networks is considerable.

## 4. Summary and Conclusions

We have conducted an adjoint sensitivity analysis with regard to changes in heat content in the Pacific Ocean using a 4D-VAR (adjoint) ocean data synthesis system. The results provide important insights into the factors influencing optimized designs of global observation schemes for enhanced ocean-state estimation on multidecadal timescales. In particular, this analysis enables us to provide useful information on effective geographical configurations for intensive deployment of Argo profiling float for the detection of the change in an important parameter associated with thermal changes in the ocean.

The sensitivities show different patterns depending on the timescale required to perform the measurements in relation to the targeted climate variations or metrics. Our results suggest that the optimal observational configuration is a sensitive function of the targeted climate variations.

Our 4D-VAR data synthesis system shows that a strategic geographical distribution of profiling floats deduced from the sensitivity analysis can potentially enhance the accuracy of estimation for a heat-content change by as much as 20% in a case.
